# Dynamic phosphorylation of Histone Deacetylase 1 by Aurora kinases during mitosis regulates zebrafish embryos development

**DOI:** 10.1038/srep30213

**Published:** 2016-07-26

**Authors:** Sara Loponte, Chiara V. Segré, Silvia Senese, Claudia Miccolo, Stefano Santaguida, Gianluca Deflorian, Simona Citro, Domenico Mattoscio, Federica Pisati, Mirjam A. Moser, Rosella Visintin, Christian Seiser, Susanna Chiocca

**Affiliations:** 1 Department of Experimental Oncology, European Institute of Oncology, Via Adamello 16, 20139 Milan, Italy; 2 The FIRC Institute for Molecular Oncology (IFOM), via Adamello 16, 20139 Milan, Italy; 3 Department of Medical Biochemistry Max F.Perutz Laboratories Medical University of Vienna, Austria

## Abstract

Histone deacetylases (HDACs) catalyze the removal of acetyl molecules from histone and non-histone substrates playing important roles in chromatin remodeling and control of gene expression. Class I HDAC1 is a critical regulator of cell cycle progression, cellular proliferation and differentiation during development; it is also regulated by many post-translational modifications (PTMs). Herein we characterize a new mitosis-specific phosphorylation of HDAC1 driven by Aurora kinases A and B. We show that this phosphorylation affects HDAC1 enzymatic activity and it is critical for the maintenance of a proper proliferative and developmental plan in a complex organism. Notably, we find that Aurora-dependent phosphorylation of HDAC1 regulates histone acetylation by modulating the expression of genes directly involved in the developing zebrafish central nervous system. Our data represent a step towards the comprehension of HDAC1 regulation by its PTM code, with important implications in unravelling its roles both in physiology and pathology.

Histone deacetylases (HDACs) are enzymes which remove acetyl moieties from ε-NH_3_ groups of lysines from both nucleosomal histones and non-histone proteins^1^; they are also known as “lysine deacetylases” (K[Lys]DAC)^2^. In humans, 18 HDACs have been identified and are divided into four classes. Class I, II and IV all include Zn^2+^-dependent enzymes^3^. Class III includes NAD^+^-dependent HDACs called Sirtuins (Sirt1-7)^4^. Class I HDACs, which include HDAC1, 2, 3 and 8 are ubiquitously expressed in all tissues^5^ and have predominantly nuclear localization. The highly related HDAC1 and HDAC2 proteins are crucial for development and physiology of the heart^6^. They are also central regulators of cell cycle progression, apoptosis and cellular proliferation. Recent evidence has shown how the fine regulation of HDAC1 and HDAC2 protein levels and the maintenance of a proper balance between the two enzymes are critical in tumour onset and progression^7^,^8^,^9^,^10^. Thus, together with HDAC2, HDAC1 is believed to be one of the master regulators of chromatin acetylation and gene expression.

HDAC1 knockout mice die at embryonic day 9.5 (E9.5)^11^. Different human cancer cell lines depleted of HDAC1 have an aberrant cell cycle, accompanied by loss of mitotic cells and an increase in apoptosis^12^. HDAC1 and HDAC2 regulate G1/S transition by binding to p21^WAF/CIP1^ and p57^Kip2^ promoters^13^,^14^, whereas inhibition of HDAC enzymatic activities by trichostatin A (TSA)^15^ or depletion of HDAC1/HDAC2 affects the G2/M progression^12^. HDAC1 and HDAC2 often display redundant roles and compensatory effects. For example, differentiation of neurons from neuronal precursors requires either HDAC1 or HDAC2^16^, while concomitant deletion of both deacetylases causes mitotic catastrophe with consequent cell death^17^. Consistently, depletion of either HDAC1 or HDAC2 in cancer cells induces a compensatory upregulation of HDAC2 or HDAC1 respectively^17^.

In zebrafish, Hdac1 is specifically required to promote neuronal specification in the developing Central Nervous System (CNS)^18^,^19^,^20^,^21^. Hdac1 is also needed for the switch from proliferation to differentiation in the zebrafish retina and optic stalk. It promotes cell cycle exit by antagonizing Notch and Wnt signaling pathways, correlating with cyclins D1 and E2 repression and leading to CDK inhibitor expression and neural progenitors cell cycle exit^20^,^21^. More in general, in zebrafish Hdac1 can function either as a positive or as a negative regulator of the cell cycle, depending on the tissue and the cell type in which it is active.

HDAC1 is a target of a complex code of post-translational modifications (PTMs, fully reviewed in ref. 22). Most recently, we have uncovered a SUMO-dependent mechanism that regulates its protein stability and expression in cancer cells^23^ and a novel phosphorylation of HDAC1 mediated by mitogens, describing a cross-talk between HDAC1 and PI3K pathways with clinical implications towards the treatment of cancer^24^. In this study we characterize an Aurora kinases-dependent, mitosis specific phosphorylation occurring on HDAC1, whose biological significance is further underscored by the fact that some viruses induce hyperphosphorylation of this HDAC at the same residues during the early phases of infection^25^,^26^,^27^. Furthermore, we provide evidence that this phosphorylation modulates the activity of HDAC1 and plays a role in regulating the proper cell cycle progression and developmental plan of zebrafish embryos. Our results contribute to elucidate how HDAC1 function may be finely regulated by a PTM, shedding light on the rationale of single HDACs regulation.

## Results

### Aurora kinases phosphorylate HDAC1 in mitosis and regulate its enzymatic activity

**Figure 1:**
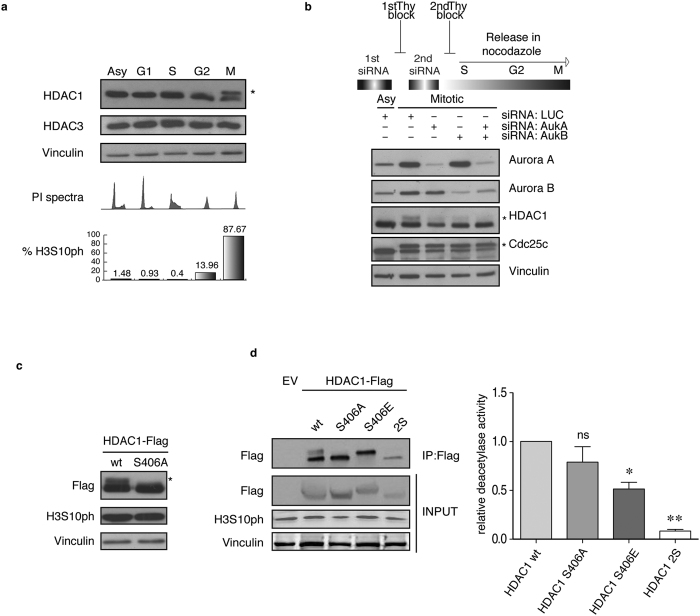
HDAC1 Aurora-dependent phosphorylation occurs specifically in mitosis and regulates its enzymatic activity. (**a**) HeLa cells were synchronized in different phases as described in Supplementary Methods and protein lysates were analyzed by western blot with the indicated antibodies. Vinculin is used as loading control. Phosphorylation levels of serine 10 of histone 3 (H3S10ph) was used as a mitotic marker and evaluated by FACS. Percentages of H3S10ph-positive cells are reported. (**b**) HeLa cells were subjected to two cycles of RNA interference with the indicated siRNA, synchronized at G1/S boundary and released with nocodazole as schematized in the cartoon. Cells were collected at 9.5 hours by mitotic shaking and analyzed by western blot with the indicated antibodies. Cdc25c phosphorylation is used as mitotic marker and Vinculin as loading control. (**c**) HeLa cells were transfected with pBJ5-HDAC1-Flag or pBJ5-HDAC1 S406A-Flag. Cells were synchronized in mitosis and analyzed by western blot with the indicated antibodies. H3S10ph is used as mitotic marker and Vinculin as loading control. (**d**) HeLa cells were transfected with 10 μg of pBJ5-HDAC-Flag various constructs as depicted and synchronized in mitosis. Lysates were used for immunoprecipitation (IP) with the Flag antibody. Three quarters of the IP were incubated with 10 μl of 3CH_3_-acetylated histones at 30 °C for 1 hour and radioactivity released was measured at the scintillation counter at count per minute (cpm); the remaining part was analyzed by Western blot. H3S10ph is used as mitotic marker, Vinculin as loading control. HDAC1 S421-423A (2S) mutant was used as the catalytically dead control^40^. The average of three independent experiments was reported as fold change versus HDAC1 wt, standard error (SEM) was indicated by the error bars; the significance was calculated by one sample t test algorithm. *P value < 0.05; **P value < 0.001; ns: not significant. In all western blots the asterisks denote the phosphorylated form of the protein.

It has been long known that HDAC1 is a critical player for the correct progression of cell cycle, but knowledge on how it is regulated remains poor. To gain insights into the molecular mechanisms, we analyzed by SDS-PAGE HDAC1 protein levels and mobility during cell cycle (Fig. 1a). HeLa cells were synchronized in different phases of the cell cycle as described in Supplementary Methods. Uniquely in mitotic cells, we observed the appearance of a slow-migrating band for HDAC1, but not for the less related class I HDAC3. Next, to better analyze the appearance of this modified form of HDAC1 as cells proceeded towards mitosis, HeLa cells were synchronized at the G1/S boundary by a double thymidine block, released in fresh medium containing nocodazole and samples were collected every hour (Supplementary Fig. S1a). The modified form of HDAC1 appeared after 7 hours, when cells started to enter mitosis and the maximum level of modification peaked at 9 hours corresponding to the maximal level of phospho-serine10-H3 (H3S10ph), a known marker of mitosis^28^. To evaluate the disappearance of the modified form of HDAC1, a purified mitotic population was released from nocodazole block, in fresh medium, to allow it to re-enter in a new G1 and samples were collected at the indicated times (Supplementary Fig. S1a). The same time-course was also performed on U2OS cells, with similar results (data not shown). The slow-migrating band of HDAC1 was likely due to a post-translational modification. It is well established that in mitosis there is a massive wave of phosphorylation for thousands of protein substrates^29^. Moreover, a previous study showed that the treatment of cells with the phosphatase inhibitor okadaic acid, which induces mitotic arrest, caused the appearance of a hyper-phosphorylated form of HDAC1^30^. We thus performed *in vitro* phosphatase assays using lambda phosphatase on extracts isolated from asynchronous and mitotic HeLa cells. As shown in Supplementary Fig. S1b, mitotic slow-migrating bands were lost after treatment with phosphatase, indicating that the slower migration of HDAC1 was indeed caused by phosphorylation. Similar results were obtained by treating mitotic samples with Antarctic phosphatase^31^ or Calf Intestinal Phosphatase^32^. Mitotic phosphorylation of HDAC1 was detected also in other cell types: 293T fibroblasts, U2OS and Saos-2 osteosarcoma cells, normal and tumoral primary human breast cells, independently from their p53 expression (data not shown) indicating that mitotic phosphorylation of HDAC1 is a general hallmark of mitosis. We thus focused on kinases activated specifically in this phase to identify the one(s) involved in this phosphorylation. Among the mitotic-restricted kinases, putative predicted consensus sites for Plk1 (Polo-like kinase 1) (serine 236 for HDAC1) and Aurora A/B kinases (serine 406 for HDAC1) were identified (Supplementary Fig. S1c). We then synchronized HeLa cells in G1/S by a thymidine block and cells were released in fresh medium for 4 hours, synchronized in mitosis by nocodazole with or without the Aurora kinase inhibitors Hesperadin or ZM-447439 or the Plk1 inhibitor BI-2536; in the last hour of treatment, we added proteasome inhibitor MG132. Mitotic cells were collected after 5.5 hours and cell lysates were analyzed. As shown (Supplementary Fig. S1d), the phosphorylated forms of HDAC1 disappeared after inhibition of Aurora kinase activity. In contrast, inhibition of Plk1 had no effect on the mitotic phosphorylation of HDAC1 but impaired the phosphorylation of its known substrate Cdc25c. Our data indicated that Aurora kinases are likely the kinases responsible for HDAC1 mitotic phosphorylation, while Plk1 had no detectable role. Moreover, partial colocalization of HDAC1 and Aurora A in mitosis had been previously reported^33^. To formally address this point, we performed RNA interference to specifically knock down Aurora A and B, alone or in combination, and looked at the effect on the mitotic phosphorylated fractions of HDAC1. HeLa cells were subjected to two cycles of RNA interference using siRNA, while concomitantly synchronizing them at the G1/S boundary by double thymidine block, as schematized in Fig. 1b. After the second block, cells were released in nocodazole containing medium to enrich for the mitotic population: mitotic phosphorylation of HDAC1 was reduced when single Aurora A or B were knocked down, with an even greater reduction when both kinases were simultaneously depleted (Fig. 1b and Supplementary Fig. S1e). These data confirmed the specific involvement of Aurora kinases in mitotic phosphorylation; moreover, they also showed that *in vivo* HDAC1 phosphorylation can be driven by both Aurora A and B kinases.

To assess whether Aurora A and B could directly modify HDAC1, we performed an *in vitro* kinase assay using recombinant Aurora A/TPX2 or Aurora B/INCENP as enzymes and HDAC1, HDAC3 or histone 3 as substrates in the presence of [^32^P]-γ-ATP. The proteins were then separated by SDS-PAGE and the gel was exposed for autoradiography (Supplementary Fig. S1f). We observed that HDAC1 is phosphorylated by both Aurora A and Aurora B, in accordance with the *in vivo* data (Fig. 1b and Supplementary Fig. S1d,e). HDAC3, as expected, was neither modified by Aurora A nor B (Supplementary Fig. S1a,d,f). By mutagenesis assays (Supplementary Fig. S1g), we then corroborated that HDAC1 is phosphorylated *in vitro* on serine 406 by both Aurora A and Aurora B. Phosphorylation of serine 406-HDAC1 (S406A) by Aurora B *in vitro* was also confirmed by mass spectrometry after *in vitro* kinase assay^31^. To further validate the identified phosphorylation sites, Flag-tagged HDAC1 wild type and HDAC1 S406A mutant were expressed in HeLa cells and analyzed during mitosis (Fig. 1c). As expected, HDAC1 wild type showed the corresponding slow-migrating phosphorylated forms, which were lost in the mutant. Furthermore, using the BT-15 antibody^31^ that specifically recognizes serine 406 phosphorylation on HDAC1 we clearly detected this modified form of HDAC1 only in untreated mitotic samples (Supplementary Fig. S1b). Finally, enzymatic activity assays carried out in mitotic cells showed reduced enzymatic activity of the phospho-mimetic form of HDAC1 S406E compared to wild type (Fig. 1d). Altogether, these data suggest that HDAC1 is phosphorylated specifically in mitosis by Aurora kinases A and B and this modification modulates the deacetylase activity of the enzyme.

### HDAC1 Aurora-dependent phosphorylation is critical for a proper developmental plan and controls the accurate cell cycle progression of zebrafish embryos

**Figure 2:**
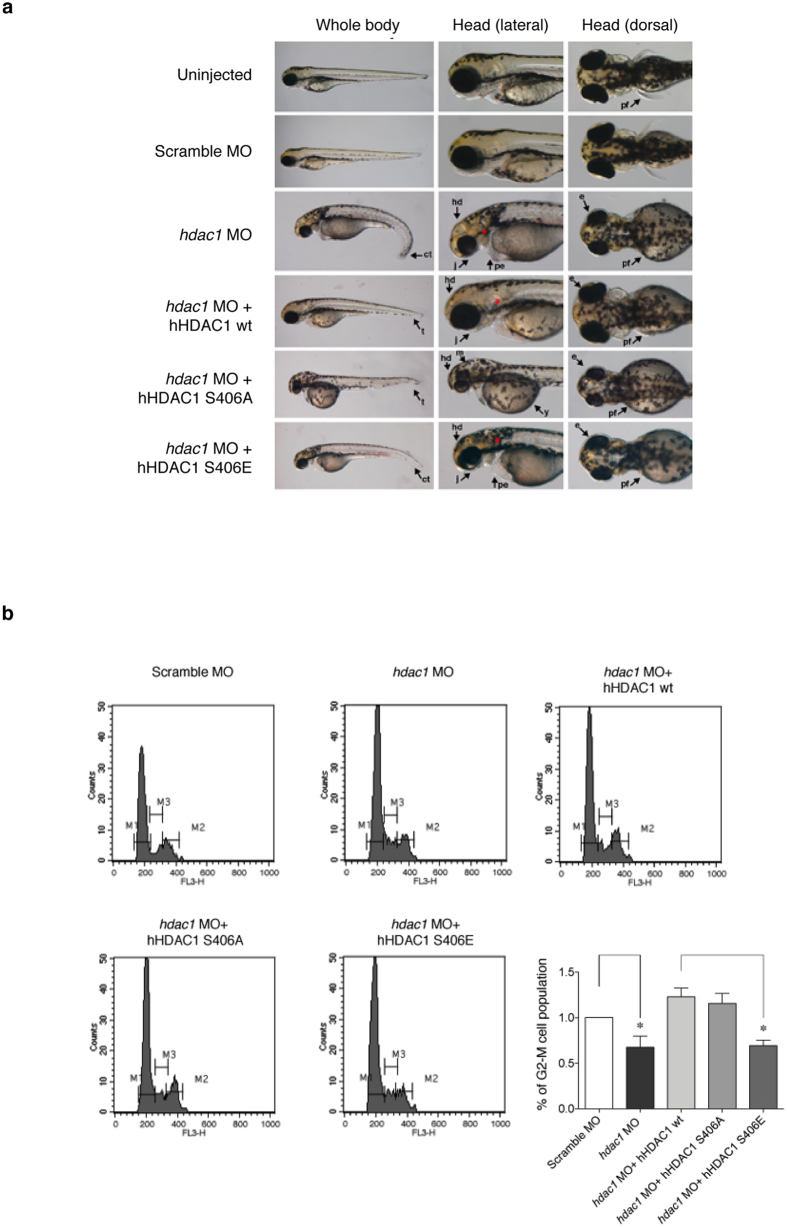
Aurora-driven phosphorylation of HDAC1 is crucial for zebrafish developmental plan and modulates proliferation in *hdac1* morphant zebrafish embryos. (**a,b**) Embryos were injected, at one cell stage, with Scramble MO or *hdac1* MO alone or in combination with hHDAC1 aurora phospho mutants and collected at 72 hours post fertilization (hpf). (**a**) Lateral and dorsal overviews of zebrafish embryos at 72 hpf. Arrows highlight morphological differences between embryos. hd: head, m: head melanocytes, j: jaw, e: eye, pe: pericardial edema, y: yolk, pf: pectoral ns, t: tail, ct: curled down tail, *: otoliths. (**b**) Flow cytometry analysis by PI staining of cell suspensions prepared from 72 hpf heads of control embryos and embryos injected at one-cell stage with *hdac1* MO alone or in combination with hHDAC1 wt, hHDAC1 S406A or hHDAC1 S406E. The percentage of G2-M cells population compared with the control is reported for every sample. The average of three independent experiments was reported, standard error (SEM) was indicated by the error bars; the significance was calculated by one sample t-test algorithm. *P value < 0.05.

In many cellular systems, due to the well-known compensation mechanism between HDAC1 and HDAC2, depletion of HDAC1 leads to an increased level of HDAC2 protein and vice versa^11^,^12^,^13^. Unlike mammals, zebrafish do not possess two distinct HDAC1 and HDAC2 genes, but they only have one, which is more similar to human HDAC1 in terms of amino acidic sequence. Perfectly coordinated and regulated cell cycles are the *conditio sine qua non* for the proper completion of a developmental program and also very slight differences likely may have dramatic impact. Moreover, many studies have pointed out the crucial role of Hdac1 during zebrafish development, in particular of the nervous system, the retina and the optical stalk^20^. Thus, to investigate the possible biological role of mitotic HDAC1 phosphorylation we used zebrafish embryos as a model. Our experimental approach consisted in knocking down the endogenous *hdac1* in zebrafish embryos right after fertilization using the morpholino strategy and reinserting the human HDAC1 wild type or Aurora phospho mutants S406A or S406E mRNA (Fig. 2a, the left panel of Supplementary Fig. S2a). We observed that the injection of a scramble morpholino did not affect the morphology of 72 hpf (hours post fertilization) embryos. On the contrary, as already known^34^, *hdac1* MO injected embryos displayed multiple developmental and morphological defects due to the absence of Hdac1: reduced size of head (hd) and jaw (j), curled down tail (ct); moreover, a pericardial edema (pe) was clearly visible. Also pectoral fins (pf) were missing, otoliths (*) were smaller and close together and melanocyte migration was defective. The expression of the human HDAC1 wt by co-injection of its mRNA together with *hdac1* MO significantly rescued the morphants phenotype: 90% of the embryos (out of 120) displayed a wild type-like phenotype with relatively normal heads, eyes and jaw structures and a straight antero-posterior body axis; reabsorption of the pericardial oedema, proper melanocyte distribution and an outline of pectoral fins are visible. Interestingly, the human Aurora phospho-null S406A mutant only partially rescued essential features of the morphant phenotype. In fact, although reduced head size, absence of pectoral fins and cell migration defects observed in morphant embryos still persisted, a proper body axis and pericardial oedema were recovered. Notably, all the body structures showed a striking developmental delay, as indicated by the late migration of the head melanocytes and yolk reabsorption. About 56% of hHDAC1 S406A injected embryos (out of 80) showed a wild type-like phenotype. Conversely, the expression of the human phospho-mimetic HDAC1 S406E was not able to rescue the phenotype in an *hdac1* morpholino background: strong defects in the development of the head and the eye were present, pericardial oedema was not reabsorbed and jaw and pectoral fins failed to form. Furthermore, there was no recovery in the body axis: a curled down tail was present and a random distribution of melanocytes was visible, particularly in the head. About 70% of the hHDAC1 S406E injected embryos (out of 70) had an *hdac1* MO-like phenotype. We verified the expression of the injected human proteins in an *hdac1* MO background (the right panel of Supplementary Fig. S2a) and using the BT-15 antibody^31^, we assessed whether also the zebrafish orthologs of the human Aurora kinases (Supplementary Fig. S2b) were able to phosphorylate hHDAC1 in zebrafish embryos. Zebrafish embryos were injected with *hdac1* morpholino alone or in combination with hHDAC1 Aurora phospho mutants, collected at 24 hpf and immunostained with BT-15 antibody. As expected, in embryos injected with a scramble morpholino no BT-15 positive cells were detected (Supplementary Fig. S3a). This was due to the high specificity of the antibody for the human form of HDAC1 (human and zebrafish HDAC1 protein differ by a 4 amino acids mismatch in the BT-15 epitope (Supplementary Fig. S2b)). Embryos co-injected with *hdac1* morpholino and hHDAC1 wt displayed a limited number of BT-15 positive cells: indeed, among all the mitotic (H3S10ph positive) cells, only prophase cells were recognized by the HDAC1 pS406 antibody (Supplementary Fig. S3a,b), as expected from our mammalian data which show HDAC1 Aurora-dependent phosphorylation in prophase^31^. Moreover, the injection of the phospho null mutant hHDAC1 S406A in a *hdac1* morpholino background completely abolished the BT-15 staining; on the contrary, the hHDAC1 S406E mutant mimicked the Aurora driven phosphorylation of the protein^31^, and therefore all the cells of the embryos injected with *hdac1* MO plus hHDAC1 S406E were BT-15 positive, independently of the phases of the cell cycle.

Given the well-established role of HDAC1 in controlling cell cycle and proliferation, we then monitored cell cycle progression at 72 hpf in embryos expressing *hdac1* MO alone or in combination with human HDAC1 Aurora-phospho mutants and in control by Propidium Iodide (PI) staining and fluorescence-activated cell sorting (FACS) analyses. Comparing cell populations of embryos heads between scramble and *hdac1* morphant embryos, we found a high and significant reduction in the G2-M populations and a corresponding increase in interphase cells (Fig. 2b). There was nearly the same change in cell cycle distribution upon co-injection of the *hdac1* MO and hHDAC1 S406E, suggesting that the phospho-mimetic hHDAC1 mutant failed to revert the cell cycle progression phenotype of *hdac1* morphant embryos. On the contrary, comparing cells from heads of control embryos and embryos co-injected with *hdac1* MO and hHDAC1wt or hHDAC1 S406A mutant, we could not detect any differences in cell cycle progression at 72 hpf. Taken together these results indicated that a proper balance of the Aurora-driven phosphorylation of HDAC1 is critical for the maintenance of a proper proliferative and developmental plan in a complex organism.

### HDAC1 Aurora-dependent phosphorylation regulates histone acetylation in *hdac1* morphant embryos and controls expression of CNS regulatory genes during zebrafish development

**Figure 3:**
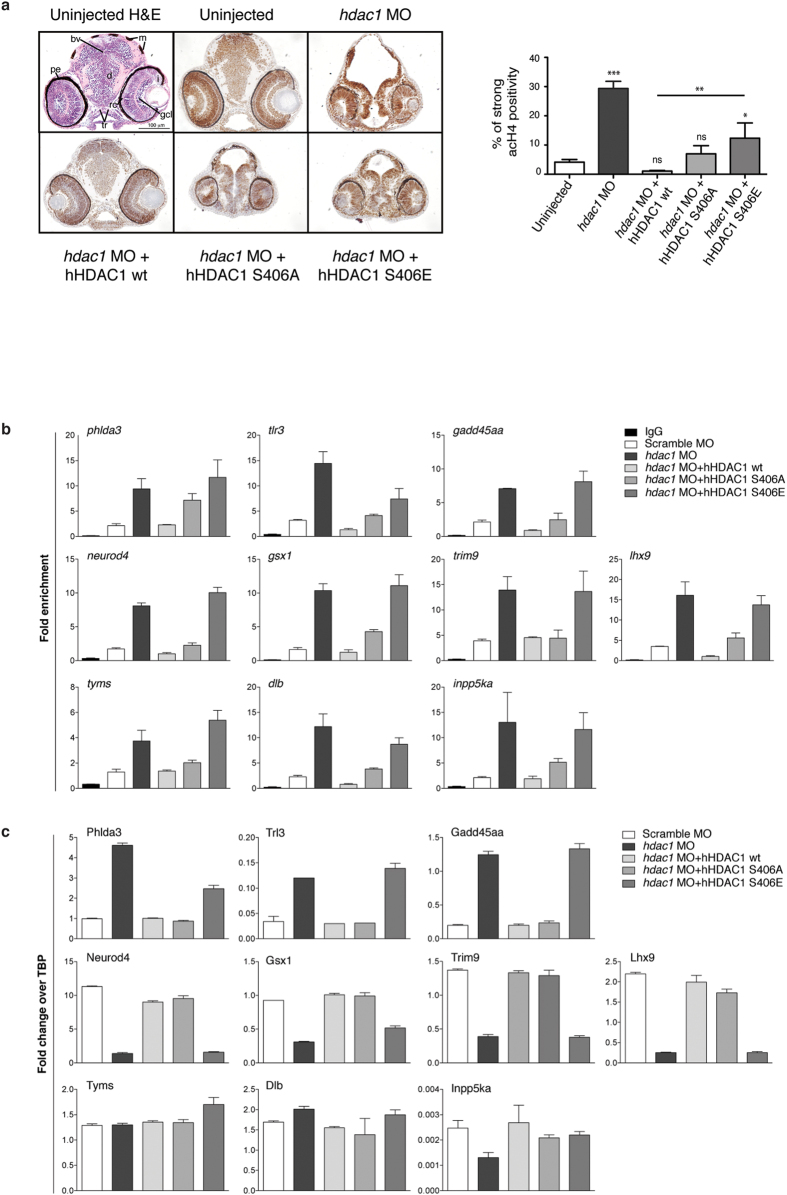
Aurora dependent phosphorylation of HDAC1 modulates histone acetylation and the expression of a core of CNS-related genes in zebrafish embryos. (**a–c**) Embryos were injected, at one cell stage, with Scramble MO (**b,c**) or *hdac1* MO alone or in combination with hHDAC1 aurora phospho mutants and collected at 72 hpf. (**a**) Immunohistochemistry staining with haematoxylin and eosin (H&E uninjected panel: d: diencephalon; pe: pigmented epithelium; tr: trabecular; rc: rods and cones; gcl: ganglion cell layer; i: iris; m: melanocytes; bv: brain ventricle) and with haematoxylin (blue) and anti-acetylated histone antibody (brown). Quantification was performed and expressed as percentage of strong positive histone H4 acetylated pixels ± SEM in the diencephalon area. All samples were compared to uninjected sample and in particular *hdac1* MO + hHDAC1 S406E was also compared to *hdac1* MO + hHDAC1 wt. **P value < 0.01; ***P value < 0.001; ns: not significant. Scale bar corresponds to 100 μm. (**b**) Chromatin immunoprecipitation (ChIP) analysis of H3K27 acetylated cis-regulatory regions of Hdac1-regulated genes. Representative data from at least two independent experiments are shown; RTqPCR data are presented as mean ± S.E.M. of three replicates. (**c**) Gene expression analysis of Hdac1-regulated genes. Representative data from three independent experiments are shown; RTqPCR data are presented as mean ± S.E.M. of three replicates. TBP is used as housekeeping.

To test whether the Aurora-dependent phosphorylation of HDAC1 is important in controlling the deacetylase activity of the enzyme *in vivo*, we performed immunohistochemistry experiments. Sections of *hdac1* morphant embryos or embryos injected with *hdac1* MO + hHDAC1 wt, S406A or S406E mutants were immunostained with (K8, K12) acetyl-Histone H4 antibody^9^,^35^ and the acetylation levels were compared to those of control embryos at 72 hpf, where there was complete abrogation of maternal *hdac1* (the left panel of Supplementary Fig. S2a). To make the data quantitatively and qualitatively comparable, for the counting of positive cells we decided to consider the same brain region of 72 hpf embryos for all samples, in particular the developing diencephalon, which is located between the eyes (Fig. 3a, denoted as “d”, and Supplementary Fig. S4). Observing the brain morphology in the sections of morphant embryos, a dramatic effect of Hdac1 KD on brain architecture is clearly visible, mainly due to an abnormal enlargement of the brain ventricles (bv) that compresses the surrounding tissues. Moreover, the analysis of embryos sections confirmed that hHDAC1 wt mRNA rescued almost completely the morphology of the brain caused by the KD of endogenous Hdac1, while the injection of the two hHDAC1 Aurora mutants mRNAs only weakly rescued Hdac1 KD (hHDAC1 S406A) or did not rescue at all the morphant phenotype (hHDAC1 S406E). As expected, knock down of endogenous Hdac1 in morphant embryos increased the percentage of strong positive histone H4 acetylated (acH4) cells compared to uninjected embryos^20^,^36^. Interestingly, we observed a significant increase in the acetylation level in the diencephalon of embryos injected with *hdac1* MO + hHDAC1 S406E in comparison to both controls (uninjected and *hdac1* MO + hHDAC1 wt), suggesting that also in an *in vivo* model the Aurora phospho-mimetic form of hHDAC1 failed to deacetylate its substrates. Conversely, embryos co-injected with *hdac1* MO + hHDAC1 wt or *hdac1* MO + hHDAC1 S406A, were able to restore the acetylation level in 72 hpf embryos brain. Control experiments were also performed to check the expression of human HDAC1 wt and mutants in injected embryos; as shown in the right panel of Supplementary Fig. S2a all three constructs were properly expressed. Notably, hHDAC1 wt expression was stronger compared to the two mutants; this might also explain the lower level of histone H4 acetylation in hHDAC1 wt samples compared to uninjected.

We then asked whether HDAC1 Aurora mutants exert their regulation by an epigenetic mechanism. To verify this hypothesis, we performed on heads of 72 hpf embryos injected at one-cell stage, with *hdac1* MO alone or in combination with hHDAC1 wt, S406A or S406E, a chromatin immunoprecipitation (ChIP) experiment with anti-acetylated lysine H3K27 antibody. The ChIP experiment was coupled to quantitative real-time PCR (RTqPCR) to evaluate the enrichment in the acetylation of the promoter of selected genes compared to their input (Supplementary Table S2). The genes used for the quantitative analysis were chosen from a list of genes involved in zebrafish developing CNS and retina, whose expression significantly changed upon *hdac1* ablation^36^. Figure 3b displays the trend of acetylated promoters of ten genes under *hdac1* knock down and/or complementation with the hHDAC1 Aurora phospho mutants. Ablation of the endogenous Hdac1 led to an increased H3K27 acetylation level of each promoter of the genes compared to the IgG control; notably, acetylated H3K27 highly increased also upon co-injection of *hdac1* MO together with hHDAC1 phospho-mimetic, supporting our previous findings of a reduced deacetylating activity for hHDAC1 S406E mutant. On the contrary, expression of hHDAC1 wt in morphant embryos completely restored the acetylation level in the promoter of the genes. To further validate our hypothesis, we checked the expression level of the genes selected for the ChIP analysis. To this aim, RNA was extracted from the heads of 72 hpf embryos injected right after fertilization with Scramble MO or *hdac1* MO alone or in combination with hHDAC1 Aurora phospho-mutants and RTqPCR were performed to measure the expression level of our subset of genes (Supplementary Table S3).

As shown in Fig. 3c, the vast majority of the genes exhibit comparable expression levels upon ablation of the endogenous *hdac1* or injection of *hdac1* MO plus hHDAC1 S406E mutant; whereas the presence of hHDAC1 wt or S406A in a morpholino background almost completely restored the expression levels of the genes. Taken together, our data indicate that HDAC1 Aurora-dependent phosphorylation is involved in the control of the catalytic activity of the enzyme both regulating histone acetylation levels and modulating expression of neurogenic regulatory genes throughout the zebrafish developing CNS.

## Discussion/Concluding Remarks

**Figure 4:**
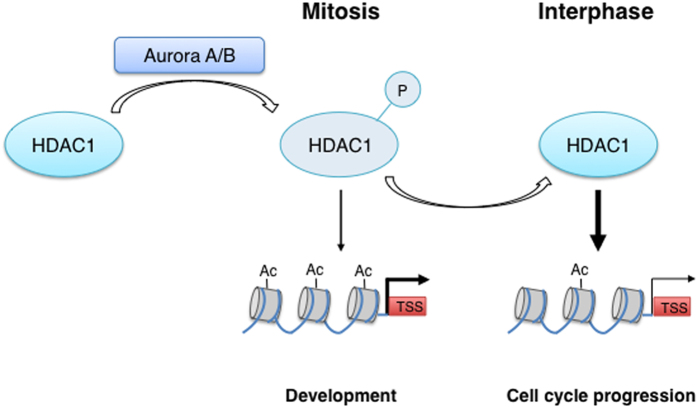
Schematic representation of our model. HDAC1 is phosphorylated by Aurora kinases A and B during prophase. The Aurora-dependent phosphorylation of HDAC1 decreases its deacetylating activity thus enhancing the expression of a subset of genes in developing zebrafish embryos and is crucial for normal development. The timely dephosphorylation of HDAC1 is required to bring the expression of target genes back to basal levels and ensure the proper progression of the cell cycle (cell cycle progression). TSS: Transcription Start Site.

Proliferation and differentiation are two interconnected pathways, especially during development, where every event of cell division must be strictly regulated in space and time to successfully complete the developmental plan. Our hypothesis is the following (Fig. 4): as cells progress from G2 to metaphase, they need to condense their chromatin, monitor the process and correct any abnormalities in chromatin architecture. HDAC1 has an important role in this process since deacetylation of key residues, such as H3K9, is a prerequisite for mitotic DNA condensation, but at a certain point HDAC1 activity must be stopped and the enzyme displaced from chromatin^32^. Our data suggest that Aurora kinases phosphorylate HDAC1 in prophase, right after cells enter mitosis. This phosphorylation, by impacting on the deacetylase activity of the enzyme, has an effect on different levels: 1) on expression level, modulating histones’ acetylation and gene expression of CNS-related genes and 2) on proliferation level, controlling proper cell cycle progression. Therefore, the Aurora-driven phosphorylation of HDAC1 could represent a further mechanism by which cells control the balance between proliferation and differentiation to ensure the inheritance of important gene signatures in the next generation of cells.

Specifically, in physiological conditions, S406 HDAC1 phosphorylation, even though very dynamically, results in transiently increased promoter acetylation of genes expressed in the CNS or with CNS-oriented functions, thus modulating their expression and enabling an accurate embryos’ development.

Thereafter, the prompt removal of the phosphate group brings promoters’ acetylation and genes’ expression levels back to their basal state. In a more exacerbated condition, i.e. mimicking HDAC1 phosphorylation by replacing the wildtype protein with HDAC1 S406E, the enzyme is constitutively less active; thereby the promoters of above mentioned genes stay highly acetylated. This situation lasting for quite a long time causes an overall morphological impairment and most importantly does not allow cells to properly go through the cell cycle. Moreover, we reasoned that it is likely that cells, which miss S406-dephosphorylated HDAC1 display a prolonged interphase due to the increased expression of factors such a Gadd45aa. It has been shown that overexpression of the mammalian homolog Gadd45 prevents G1/S transition^37^,^38^. Alternatively or in addition, histone hyperacetylation in the absence of fully active S406-dephosphorylated HDAC1 might directly interfere with normal S-phase transition given the known role of HDAC1 in DNA replication^39^.

On the contrary, the absolute abrogation of the HDAC1 Aurora-dependent phosphorylation does not affect proper cell cycle progression, but embryos display a clear developmental delay probably due to the fact that expression of genes persists at basal levels. In conclusion, we hypothesize that while controlling the deacetylase activity of HDAC1, the Aurora-dependent phosphorylation on one hand modulates the expression of genes directly involved in zebrafish development, and, on the other hand, acts as sensor of the transcriptional process, regulating in a fine-tuned manner proper cell cycle progression.

## Experimental Procedures

### Zebrafish strains and maintenance

Zebrafish strains were maintained and bred according to standard procedures. Embryos from AB wild type strain were maintained in E3 water (5 mM NaCl, 0.17 mM KCl, 0.33 mM CaCl_2_, 0.33 mM MgSO_4_) at 28.5 °C.

### Ethics statement

Fish were maintained/raised according to EU regulations on laboratory animals.

All experimental protocols (project number 02/14) were approved by the Institutional Animal Care and Use Committee (IACUC) of IFOM (FIRC Institute of Molecular Oncology, Via Adamello 16, 20139, Milan Italy) and by the Italian Ministry of Health.

### mRNAs and morpholino injections in zebrafish embryos

mRNAs were synthesized from NotI digested pCS2+hHDAC1 wt, S406A, S406E plasmids using mMessage mMachine kit (Ambion) and purified with Microcon YM-100 (Millipore) filter devices. RNA quality was assayed by means of gel electrophoresis. mRNAs was then diluted in 1X Danieau solution (58 mM NaCl, 0.7 mM KCl, 0.4 mM MgSO_4_, 0.6 mM Ca(NO_3_)_2_, 5 mM HEPES, pH 7.6) at final concentration of 120 ng/μl and pressure injected into 1–2 cell stage embryos.

Morpholinos were purchased by Gene Tools, LLC. The morpholino against *hdac1* (5′-TTGTTCCTTGAGAACTCAGCGCCAT-3′) was targeted to the translational initiation site, as ref. 19. The scramble morpholino sequences was: 5′-CCTCTTACCTCAGTTACAATTTATA-3′. Morpholinos (0,15 mM) were diluted and injected as described above.

### Chromatin immunoprecipitation (ChIP) and quantitative real-time PCR (RTqPCR)

Fixed head embryos cells were lysed in RIPA buffer and after chromatin shearing by sonication, incubated at 4 °C overnight with protein G Dynabeads (Invitrogen) and anti-acetylated H3K27 antibody (Abcam ab4729). Extended methods are in the Supplementary Information.

## Additional Information

**How to cite this article**: Loponte, S. *et al*. Dynamic phosphorylation of Histone Deacetylase 1 by Aurora kinases during mitosis regulates zebrafish embryos development. *Sci. Rep*. **6**, 30213; doi: 10.1038/srep30213 (2016).

## Supplementary Material

Supplementary Information
