# Supplementary material for: Dynamic phosphorylation of Histone Deacetylase 1 by Aurora kinases during mitosis regulates zebrafish embryos development
Source: Sci Rep. 2016 Jul 26;6:30213. doi: 10.1038/srep30213 (PMC4960611; doi:10.1038/srep30213)
Supplement: Supplementary Information [file srep30213-s1.pdf]

Supplementary Information

Dynamic phosphorylation of Histone Deacetylase 1 by Aurora kinases during mitosis regulates zebrafish embryos development

Running title: **Mitotic phosphorylation of HDAC1 by Aurora kinases**

Sara Loponte*[1], Chiara V. Segré*[1,2], Silvia Senese*[1,3], Claudia Miccolo*[1], Stefano Santaguida[1,4], Gianluca Deflorian[5], Simona Citro[1], Domenico Mattoscio[1], Federica Pisati[5], Mirjam Moser [6], Rosella Visintin[1], Christian Seiser [6] and Susanna Chiocca [1#]

[1] Department of Experimental Oncology, European Institute of Oncology, Via Adamello 16, 20139 Milan, Italy; [2] Present address: Fondazione Umberto Veronesi, Piazza Velasca 5, 20121 Milano, Italy; [3] Present address: Department of Chemistry and Biochemistry, University of California, Los Angeles, Los Angeles, CA 90095, USA; [4] Present address: Koch Institute for Integrative Cancer Research at MIT, Massachusetts Institute of Technology, Cambridge, USA; [5] The FIRC Institute for Molecular Oncology (IFOM), via Adamello 16, 20139 Milan, Italy; [6] Max F. Perutz Laboratories, Medical University of Vienna, Austria

*These authors equally contributed to the work

[#] Corresponding author: susanna.chiocca@ieo.eu

Supplementary Figure 1. Aurora kinases A and B phosphorylate HDAC1 in mitosis on serine 406 *in vitro*

(**a**) HeLa cells were synchronized at the G1/S boundary or in mitosis as described in Supplementary Methods. Samples were collected every hour and analyzed by western blot with the indicated antibodies. RbAP48 is used as loading control. Cell synchronization was evaluated by FACS. Asterisks indicate the slow-migrating, modified forms of HDAC1.

(**b**) λ -phosphatase assay on asynchronous and mitotic HeLa cells. The asterisk indicates the hyper-phosphorylated forms of HDAC1. BT-15 recognizes HDAC1 mitotic phosphorylation (Segre' et al., 2016). Cdc25c is used as positive control for the assay, Cyclin B1 as mitotic marker, Vinculin as loading control.

(c) Alignment of human class I HDAC1 (GeneBank CAG_46518), HDAC2 (NCBI NP_001518) and HDAC3 (NCBI NP_003874) protein sequences. Putative target sites identifed by the Phosida bioinformatic tool for Aurora kinases (pink) and Plk1 (green) are indicated. Numbers of target residues are referred to the HDAC1 sequence.

(**d**) HeLa cells were synchronized in G1/S by thymidine block and released in fresh medium for 4 hours, synchronised in G2/M phase by nocodazole with or without the Aurora kinase inhibitors Hesperadin and ZM-447439 or the Plk1 inhibitor BI-2536; in the last 1 hour of treatment, we added proteasome inhibitor MG132. Mitotic cells were collected after 5.5 hours by mitotic shaking and analyzed by western blot with the indicated antibodies. Cdc25c is a known substrate of Plk1 in mitosis and here is used as positive control for BI2536. H3S10ph is a known substrate of Aurora kinases and here is used as positive control of ZM-447439 and Hesperadin effects. Cyclin B1 is used as marker of mitosis and Vinculin as loading control.

(**e**) HeLa cells were subjected to two cycles of RNA interference with the indicated siRNA, synchronized at G1/S boundary and released with nocodazole as schematized in the cartoon. Cells were collected at 9.5 hours by mitotic shaking and analyzed by western blot with the

indicated antibodies. BT-15 recognizes HDAC1 mitotic phosphorylation (Segre' et al., 2016). Cdc25c phosphorylation is used as mitotic marker and Vinculin as loading control. Densitometric analysis was performed using Image J and the average of two independent experiments was indicated ± SEM.

(**f**) Recombinant Aurora A/TPX2 and Aurora B/INCENP were incubated with 1 μg of HDAC1, HDAC3 or histone 3 (H3) as substrates with 5 μCi ATP (γ32P) for 1 hour at 30°C. Samples were loaded on a 14% PAA gel and incubated for autoradiography. H3 is used as a positive control for Aurora kinase activity. Coomassie-stained gels are reported as loading controls. Asterisks mark the bands corresponding to recombinant proteins. A ladder of molecular weight is reported, where KDa are kilo Daltons.

(**g**) Recombinant Aurora A/TPX2 and Aurora B/INCENP were incubated with 1μg of HDAC1 wild type and the indicated serine-to-alanine mutants as substrates with 5μCi ATP(^{32}P) for 1 hour at 30°C. Samples were loaded on a 14% gel and incubated for autoradiography. Coomassie-stained gels are shown as loading controls.

Supplementary Figure 2. Overexpression of hHDAC1 in zebrafish embryos

(**a**) Western blot analysis of protein extracts from control embryos or Hdac1 morphant embryos. On the left samples were collected at 24, 48 or 72 hpf and blotted with zfHdac1; asterisk indicates the zfHDAC1 band. On the right samples were collected at 72 hpf and blotted with Flag. Anti-Histone3 (H3) antibody was used as a loading control.

(**b**) The BT-15 epitope recognizing the KRISI Aurora consensus motives of HDAC1 is reported. The non-conserved residues between human and zebrafish HDAC1 are shown. The amino acids are indicated according to the universal one letter code.

Supplementary Figure 3. Aurora kinases phosphorylate hHDAC1 in zebrafish embryos

(**a**) Immunofluorescence microscopy of 24 hpf zebrafish embryos. Embryos were injected at one-cell stage with scramble MO or *hdac1* MO alone and in combination with hHDAC1 wt, S406A or S406E mutants, collected at 24 hpf, fixed in 4% paraformaldehyde and then immunostained with the specific antibody. Scale bar corresponds to 50 μm.

(**b**) Immunofluorescence microscopy of zebrafish embryos injected at one-cell stage with *hdac1* MO and hHDAC1 wt, collected at gastrulae stage (80% epiboly) and stained with the indicated antibody. Scale bar corresponds to 100 μm.

Supplementary Figure 4. Detailed quantification of the H4 acetyl signal in 72 hpf uninjected embryos (from Fig. 3a Uninjected panel).

Left Panel: Immunohistochemistry staining of 72 hpf uninjected embryos (from Figure 3a) with haematoxylin (blue) and acetylated-histone H4 antibody (clone T25) (brown) (Ronzoni et al., 2005). Right Panel: The diencephalon area was identified with a pink line and used for the analysis. Aperio ImageScope software, used for quantification, identified blue pixel as negative, yellow pixels as weak positive, orange pixels as positive and red pixels as strong positive acetylated-histone H4. The graph expresses the distribution of total pixels as percentage.

Supplementary Table 1. PCR site-directed mutagenesis primer sequences

5′-3′ Forward and reverse primer sequences are reported

**HDAC1 KRISI S406A**	CCCTGACAAGCGCATCGCGATCTGCTCCTCTGAC
	GTCAGAGGAGCAGATCGCGATGCGCTTGTCAGGG
**HDAC1 KRISI S406E**	CCCTGACAAGCGCATCGAGATCTGCTCCTCTGAC
	GTCAGAGGAGCAGATCTCGATGCGCTTGTCAGGG
**HDAC1 S421/423°**	GTGAGGAAGAGTTCGCCGATGCGGAAGAGGAGGGAGAGG
	CCTCTCCCTCCTCTTCCGCATCGGCGAACTCTTCCTCAC
**HDAC1 S434A**	GGGGGCCGCAAGAACGCTTCCAACTTCAAAAAAGCC

	GGCTTTTTTGAAGTTGGAAGCGTTCTTGCGGCCCCC
**HDAC1 S435A**	GGTGGTCGCAAGAACTCTGCTAACTTCAAAAAAGCC
	GGCTTTTTTGAAGTTAGCAGAGTTCTTGCGACCACC
**HDAC1 T445A**	GCCAAAAGAGTTAAAGCAGAGGATGAGAAAGAGAAAGATCC
	GGATCTTTCTCTTTCTCATCCTCTGCTTTAACTCTTTTGGC

Supplementary Table 2. ChIP primer sequences

5′-3′ Forward and reverse primer sequences are reported for every target. Primer sequences are designed on the promoter region. *Desert region* corresponds to chr1:9310554+9310633.

trim9	TGCTGATGATGCGAAGTGTG
	TAGGAAAATCAGCCGCGTTG
phlda3	CCTCTGTGGCCAATGATCAA
	CCAACATGCCCAGATTACGG
inpp5ka	GGCTGCGGGGAAGTTAAAAT
	GCCTTTCTGTTACTGTTCAAGC
neurod4	GTCTGAGGGAGTTTGCCTGT
	GCTGAGTTTCTTACGACGCA
gsx1	CCATGTGATCGCGTGAGAAA
	GTACTGCCCCTTCTCCCTTT
gadd45aa	GGCGCGAAAACCAATCAAAG
	GACTTTGCGAGGCGTTACAT
tyms	GGTGGCGGTATGGACTTTTC
	CTTTTGCCGCGAAACTGTTC
tlr3	ACATGGATTGTTTGGGCATT
	TCAATCAAATAAGCCGTCCA
dlb	CATCAACACCACTGCCACAT
	CGTGACGTCAGAGGTGACATA
lhx9	TCTTCAAACCGATGGTGATG
	TCAAAGTGGCAAAACACCAG
desert region	TTGAAGATGTTCAGCGTTGG
	TGGCTGAAACACCAATGAAA

Supplementary Table 3. Gene expression primer sequences

5′-3′ Forward and reverse primer sequences are reported for every gene.

**Dlb**	TGTAGCCACAAACCATGTGC
	GCTGCAAAGTCCCTCAATGT
**Gadd45aa**	CCAGATCCACGACAGTTCC
	ACTCTCTCCCCGTTGCATTA
**Gsx1**	TCTCTGGGGATCATATTGAAGG
	GGCTCTCTGAACACCCATTT
**Inpp5ka**	GAAGGAGACGTCGAACTTTGAG
	CAAACTTGATTTGAAGACGATAGC
**Lhx9**	TCGGGTTCTGATTCTCTCATC
	GGAGTACTGTCTGGAGGTTATCAAA
**Neurod4**	GGAGCCGAATACAAAGATATGC
	CCGTCATATGGACCCGTCT
**Phlda3**	CATCCACACTCTCTGTATTTTGGA
	GACCTGAATGTCTCTGATGAAGG
**Tbp**	GGTTTTCCGTAGGGTTTGGT
	GTGCTGGTGAACGCAGTG
**Tlr3**	CTTCACATCGTCGTCGTCA
	TGGGTTTGACTTGATGGTGTC
**Trim9**	TGTATCGATAGCCTTGAAACACC
	GAGAGCCGCTGAAACCAG
**Tyms**	GCTCATGGAGGGCATAGTTG
	ATGATGACGGCAGCAAAAC
**Tbp**	CAAACTTGATTTGAAGACGATAGC
	ATGATGACGGCAGCAAAAC

Supplementary Methods

Cell lines and culture conditions

HeLa cells were maintained in Dulbecco’s modified Eagle’s medium (Lonza) supplemented with 10% fetal bovine serum (Lonza), 2 mM L-glutamine (Lonza) and antibiotics (250 μg/ml penicillin and 25 μg/ml streptomycin). Cells were cultured in a humidified 37°C incubator with 5% CO2.

Cell synchronization

Cells were synchronized at the G1/S boundary by double thymidine block: cells were treated with 2 mM thymidine overnight, followed by wash and release in fresh medium, for two consecutive nights. To synchronize cells in mitosis, the second day, they were released in medium containing 330 nM nocodazole for 9.5 hours and mitotic, rounded-up cells were harvested by shaking them off culture dishes. The G2-enriched population is the one that remains attached to the plate upon nocodazole treatment and mitotic shaking. G1 pure population was obtained from mitotic cells released for 2 hours from nocodazole block in order to re-enter synchronously in a new G1.

In vitro phosphatase assays

Cells were lysed in JLB buffer (50 mM Tris pH 8, 150 mM NaCl, 10% glycerol, 0.5% Triton X-100, PMSF, aprotinin and leupeptin) and λ-phosphatase (NewEngland Biolabs) was added to 50 µg of protein lysates. The reaction was carried on at 30°C for 1 hour.

Production and purification of GST tag proteins

GST tag proteins were produced in bacteria and purified with Glutathion® beads (Sigma). Proteins were eluted using reduced glutathione and the GST tag was removed through proteolytic cleavage with PreScission protease (produced as recombinant protein at our campus facilities).

Quantification of purified recombinant protein was performed by SDS-PAGE and gel staining with Coomassie Brilliant Blue, by comparison with known amounts of BSA protein.

In vitro Aurora kinase assay

Aurora kinase assays were performed as previously described (Santaguida et al., 2010). Briefly, 1 μg of purified substrate (HDAC1, HDAC3 or histone H3) was incubated with 50 ng of recombinant-purified Aurora A/TBX2 or Aurora B/INCEP kinase in the presence of 5 μCi of γ-32pATP at 30°C for 1 hour and analyzed by SDS-PAGE followed by autoradiography.

In vitro histone deacetylase assay

Flag-tagged HDACs were transfected in cells and immune-precipitated. Purified complexes were then incubated with 100 μCi of chicken purified H^3-acetyl histones in E1A buffer [(50 mM Hepes pH 7.5, 250 mM NaCl, 0.1% NP-40) PMSF, leupeptin, aprotinin, sodium orthovanadate, sodium fluoride and NEM were freshly added immediately before use] for 1 hour at 30°C. H^3-acetyl groups released were extracted with a two-phase acqueous-ethyl acetate solution and radioactivity measured as counts per minute (c.p.m.) in a ß-counter scintillation machine.

For the enzymatic activity, the scintillation counts were normalized versus the amount of immunoprecipitated proteins.

Plasmids and vectors

For expression in human cells the pBJ5-HDAC1-Flag was used. For expression of recombinant proteins in bacteria the pGEX-6P1-GST-HDAC1 and pGEX-6P1-GST-HDAC3 plasmids were used. Plasmids for production of Aurora kinases have been previously described (Santaguida et al., 2010; Sessa et al., 2005). Point mutant HDAC1 proteins were generated by PCR site directed mutagenesis of pBJ5-HDAC1 wild type using primers carrying the desired mutations (see **Supplementary Table 1**).

For mRNA transcription and injection in zebrafish embryos, the human HDAC1 wild type, S406A and S406E constructs were subcloned into pCS2+ expression vector (Addgene) using BamHI/XhoI restriction enzymes.

Western blot analysis

For western blot analysis, cells were lysed in denaturing SDS lysis buffer [one volume of Buffer I (5% SDS, 150 mM Tris-HCl pH 6.8, 30% glycerol) + three volumes of Buffer II (25 mM Tris-HCl pH 8.3, 50 mM NaCl, 0.5% NP-40, 0.5% deoxycholate, 0.1% SDS) + aprotinin and leupeptin, sodium fluoride, sodium pyrophosphate and sodium orthovanadate].

For HDAC1 detection during zebrafish embryogenesis, groups of 80 dechorionated embryos of 24, 48 and 72 hpf (24 and 48 hpf embryos were dechorionated with 1 mg/ml Pronase solution) were washed 2 times with ½ Ginzburg fish-ringer solution (NaCl 56 mM, KCl 1.6 mM; $CaCl_2$ 1.3 mM, $NaHCO_3$ 1.2 mM) in order to remove their yolk and lysed in urea buffer (8 M Urea, 0.1 M NaH2PO4, 0.01 M Tris, pH 8).

The following antibodies were used for western blot: HDAC1 mouse, clone 10E2 produced in house by Christian Seiser, HDAC1 rabbit (Abcam ab7028), HDAC3 rabbit (Abcam ab7030), Vinculin mouse (Sigma v9113), pS406-HDAC1 mouse (clone BT-15, Segre' et al., 2016), histone H3 rabbit (Abcam ab1791), RbAp48 mouse (Abcam ab55778), H3S10ph rabbit (Upstate 06-570), Cdc25c rabbit (Santa Cruz sc-327), Cyclin B1 mouse (Santa Cruz sc-166757), Flag rabbit (Sigma f3136), zebrafish HDAC1 rabbit (Abcam ab41407).

Immunoprecipitations

Cells were lysed in non-denaturing E1A buffer [(50 mM Hepes pH 7.5, 250 mM NaCl, 0.1% NP-40) PMSF, leupeptin, aprotinin, sodium orthovanadate, sodium fluoride and NEM were freshly added immediately before use]. Immunoprecipitation (IP) was performed incubating

protein extracts with the antibody of interest at ratio of 3-4 mg Ab/mg of crude extract for 16 hours at 4 °C on rotation. Then protein-A/sepharose-beads (slurry 50%) were added to the samples for 2 hours at 4 °C. Beads were then extensively washed with cold E1A buffer, loaded on SDS-PAGE and analyzed by western blot with the indicated antibodies. Total extracts (input) were loaded as a control.

Zebrafish embryo cell dissociation

Embryos were anesthetized with tricaine and decapitated. After transient storage in PBS on ice, heads were incubated in trypsin-EDTA (Lonza) for 30 min at 37°C. Afterward, heads were transferred to fresh PBS and mechanically disgregated. This treatment led to a single-cell suspension. Cells were harvested by centrifugation for 3 min at 3000 × g at 4°C, resuspended in PBS and used for further experiments.

FACS analysis

For FACS analysis, cell suspension obtained as above mentioned was fixed in ice-cold 70% ethanol and incubated over night on ice. DNA was stained with a solution containing 2.5 µg/ml propidium iodide (PI) (Sigma) and 250 µg/ml RNase A. Samples were acquired using a FACSCalibur flow cytometer (Becton Dickinson), and data were analyzed using CellQuest software (Becton Dickinson).

Chromatin immunoprecipitation (ChIP) and quantitative real-time PCR (RTqPCR)

For each batch of chromatin, 70 wild type (AB), hHDAC1 mutants, Scramble MO or *hdac1* morphant embryos were enzymatically dechorionated at 72 hpf, anesthetized with tricaine and decapitated. Embryos' heads were dissociated and fixed immediately in 1.5% formaldehyde

for 10 minutes. Glycine (2.5 M) was added to quench the formaldehyde and cell suspension was washed in ice cold PBS and then lysed in RIPA buffer [(10 mM Tris-HCl pH 8.0, 1 mM EDTA pH 8.0, 140 mM NaCl, 1% Triton X-100, 0.1% SDS, 0.1% Na-Deoxycholate) PMSF, leupeptin, aprotinin, sodium orthovanadate, sodium fluoride were added before use]. Chromatin was sheared by sonication to give DNA fragments of approximately 300-700 bp in size. Sonicated samples were centrifuged at 12000 × g at 4°C for 15 minutes, and insoluble material was discarded. The supernatant was incubated with acetylated H3K27 antibody (Abcam) or control IgG antibody (Abcam). Dynabeads Protein G magnetic beads (Invitrogen) were added to each sample and the samples rotated at 4°C overnight. Beads were washed six times with RIPA washing buffer (50 mM Hepes-KOH pH 7.5, 500 mM LiCl, 1 mM EDTA, 1% NP-40, 0.7% Na-Deoxycholate) at 4°C and bound complexes were eluted from the beads in Elution Buffer (1x TE, 2% SDS) shaking for 20 minutes at 65°C. Cross-links were reversed overnight at 65°C and DNA fragments were purified with Qiaquick purification kit (Qiagen). Quantitative real-time PCR (RTqPCR) of immuno-precipitated DNA was used to validate ChIP experiment. RTqPCR analyses were performed in triplicate and DNA abundance was normalised against Input values.

RNA extraction

Embryos were anesthetized with tricaine and decapitated. After transient storage in PBS on ice, heads were disaggregated in TRIzol® and RNA was extracted following the manufacturing instructions (TRI Reagent® Protocol/ Sigma-Aldrich).

Confocal immunofluorescence microscopy

For immunostaining, gastrulae or 24 hpf embryos were fixed in 4% para-formaldehyde (PFA) overnight and permeabilized with acetone at -20°C for 7 minutes. Embryos were then blocked

in PBS containing 0.5% Triton-X, 1% DMSO, 1% BSA and 2% sheep serum (PBST) for 2 hours at 4°C and incubated with primary antibody overnight. The next day, embryos were rinsed in PBST and incubated with secondary antibody. Then, embryos were stained with DAPI before mounting for confocal microscopy.

The following primary antibodies were used: pS406-HDAC1 (clone BT-15, Segre et al., 2016), H3S10ph (Upstate).

The following secondary antibodies were used: Alexa-488 conjugated mouse IgG, Alexa-433 conjugated rabbit IgG.

Immunohistochemistry (IHC)

For histological sections, 72 hpf embryos were fixed in 4% PFA overnight at 4°C and embedded in 1.2% low-melting agarose: while the agarose solidify, embryos were properly oriented. Then, the embryos-containing agarose blocks were dehidated and included in paraffin before sectioned with microtome. 5μm transversal sections of the heads of the embryos were dehydrated and incubated with Na-citrate buffer (10mM dihydrated Na-citrate pH6, 0,05% Tween20) at 95 °C for 45 minutes, to unmask the antigen from the paraffin. Later they were blocked for 1 hour with blocking solution (PBS, 1% BSA, 2% FBS) and incubated with primary antibody overnight. The day after, sections were washed in PBS and incubated with secondary antibody. The antibody signal was revealed with DAB and haematoxylin staining was performed, then sections were dehydrated and mounting with Eukitt® mounting medium.

For immunohistochemistry, acetylated-histone H4 antibody (clone T25) was used (Ronzoni et al., 2005).

The percentage of acetylated histones (acH4) strong positive pixels was calculated using Aperio ImageScope software and is reported for every sample as average of at least three

independent experiments, Statistical differences were evaluated using Newman-Keuls multicomparison (ANOVA) analysis.

Supplementary References

Ronzoni, S., Faretta, M., Ballarini, M., Pelicci, P., and Minucci, S. (2005). New method to detect histone acetylation levels by flow cytometry. Cytometry A *66*, 52-61.

Santaguida, S., Tighe, A., D'Alise, A.M., Taylor, S.S., and Musacchio, A. (2010). Dissecting the role of MPS1 in chromosome biorientation and the spindle checkpoint through the small molecule inhibitor reversine. J Cell Biol *190*, 73-87.

Segré CV, Senese S, Loponte S, Santaguida S, Soffientini P, Grigorean G, Cinquanta M, Ossolengo G, Seiser C, Chiocca S. (2016). A monoclonal antibody specific for prophase phosphorylation of histone deacetylase 1: a readout for early mitotic cells.
MAbs. Jan 2;8(1):37-42. doi: 10.1080/19420862.2015.1098795. Epub 2015 Oct 14.

Sessa, F., Mapelli, M., Ciferri, C., Tarricone, C., Areces, L.B., Schneider, T.R., Stukenberg, P.T., and Musacchio, A. (2005). Mechanism of Aurora B activation by INCENP and inhibition by hesperadin. Mol Cell *18*, 379-391.

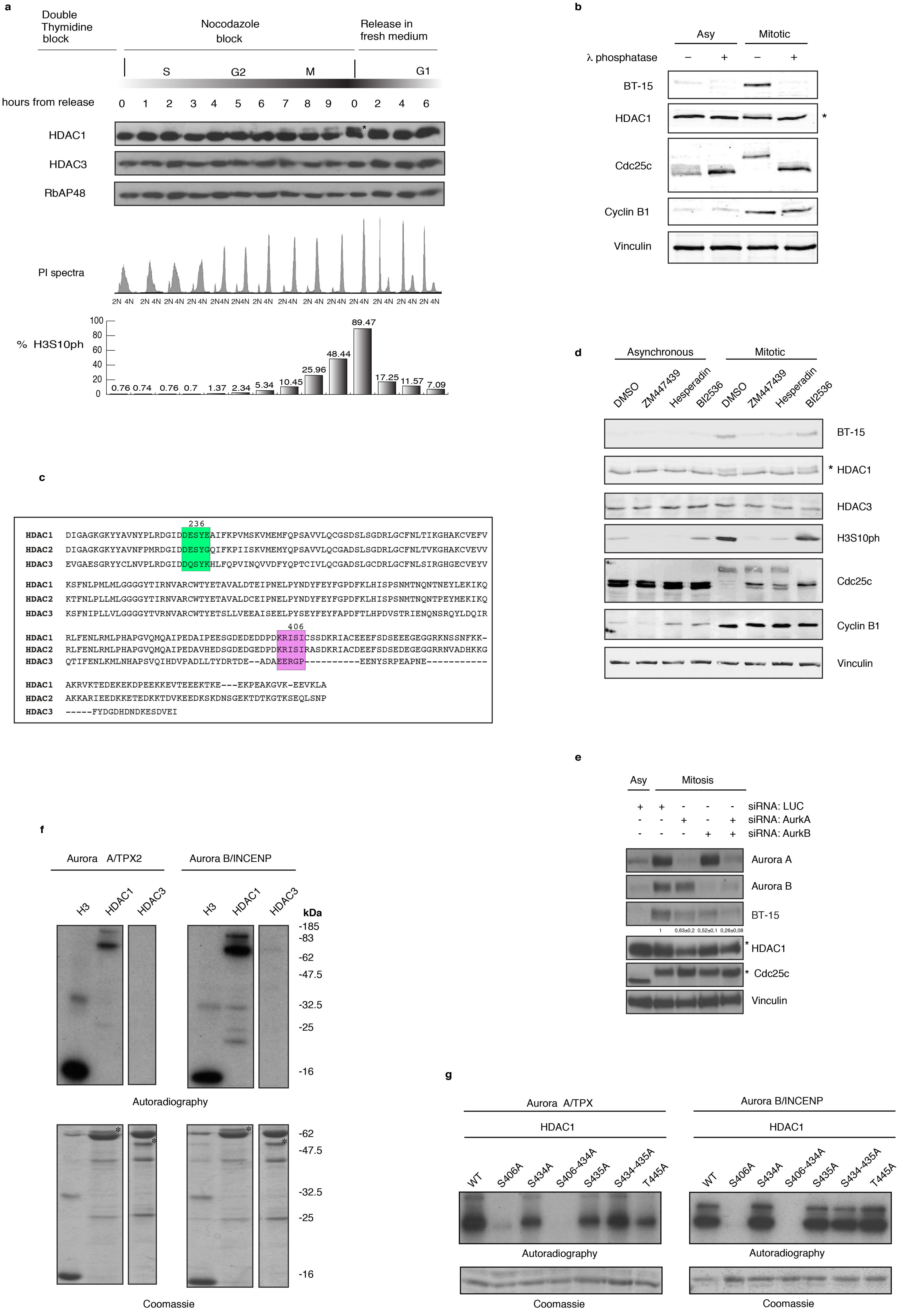
a
Double Thymidine block
Nocodazole block
Release in fresh medium
S
G2
M
G1
hours from release
0 1 2 3 4 5 6 7 8 9 0 2 4 6
HDAC1
HDAC3
RbAP48
PI spectra
2N 4N
% H3S10ph
100
80
60
40
20
0
0.76 0.74 0.76 0.7 1.37 2.34 5.34 10.45 25.96 48.44 89.47 17.25 11.57 7.09
b
Asy
Mitotic
λ phosphatase
BT-15
HDAC1
Cdc25c
Cyclin B1
Vinculin
c
236
HDAC1 DIGAGKGKYYAVNYPLRDGIDDESYEAIFKPVMSKVMEMFQPSAVVLQCGSDSLSGDRLGCFNLTIKGHAKCVEFV
HDAC2 DIGAGKGKYYAVNFPMRDGIDDESYGQIFKPIISKVMEMYQPSAVVLQCGADSLSGDRLGCFNLTVKGHAKCVEVV
HDAC3 EVGAESGRYYCLNVPLRDGIDDQSYKHLFQPVINQVVDFYQPTCIVLQCGADSLGCDRLGCFNLSIRGHGECVEYV
HDAC1 KSFNLPMLMLGGGGYTIRNVARCWTYETAVALDTEIPNELPYNDYFEYFGPDFKLHISPSNMTNQNTNEYLEKIKQ
HDAC2 KTFNLPLLMLGGGGYTIRNVARCWTYETAVALDCEIPNELPYNDYFEYFGPDFKLHISPSNMTNQNTPEYMEKIKQ
HDAC3 KSFNIPLLVLGGGGYTVRNVARCWTYETSLLVEEAISEELPYSEYFEYFAPDFTLHPDVSTRIENQNSRQYLDQIR
406
HDAC1 RLFENLRMLPHAPGVQMQAIPEDAIPEESGDEDEDDPDKRISICSSDKRIACEEEFSDSEEEGEGGRKNSSNFKK-
HDAC2 RLFENLRMLPHAPGVQMQAIPEDAVHEDSGDEDGEDPDKRISIRASDKRIACDEEFSDSEDEGEGGRRNVADHKKG
HDAC3 QTIFENLKMLNHAPSVQIHDVPADLLTYDRTDE--ADAEERGP----------EENYSRPEAPNE-----------
HDAC1 AKRVKTEDEKEKDPEEKKEVTEEEKTKE---EKPEAKGVK-EEVKLA
HDAC2 AKKARIEEDKKETEDKKTDVKEEDKSKDNSGEKTDTKGTKSEQLSNP
HDAC3 -----FYDGDHDNDKESDVEI
d
Asynchronous
Mitotic
DMSO
ZM447439
Hesperadin
BI2536
BT-15
HDAC1
HDAC3
H3S10ph
Cdc25c
Cyclin B1
Vinculin
e
Asy
Mitosis
siRNA: LUC
siRNA: AurkA
siRNA: AurkB
Aurora A
Aurora B
BT-15
1
0,63±0,2
0,52±0,1
0,26±0,08
HDAC1
Cdc25c
Vinculin
f
Aurora A/TPX2
Aurora B/INCENP
H3
HDAC1
HDAC3
kDa
-185
-83
-62
-47.5
-32.5
-25
-16
Autoradiography
Coomassie
g
Aurora A/TPX
Aurora B/INCENP
HDAC1
WT
S406A
S434A
S406-434A
S435A
S434-435A
T445A
Autoradiography
Coomassie

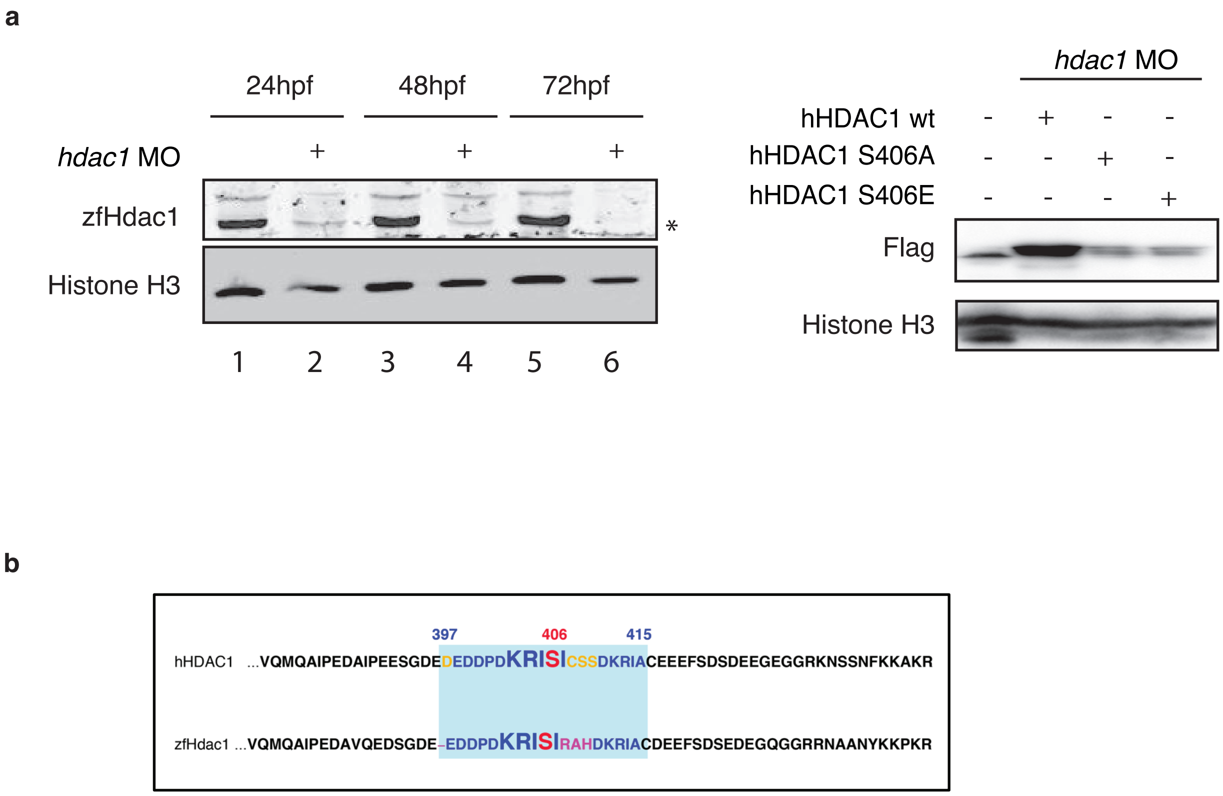
a
24hpf
48hpf
72hpf
hdac1 MO
+
+
+
zfHdac1
*
Histone H3
1
2
3
4
5
6
hdac1 MO
hHDAC1 wt
- + - -
hHDAC1 S406A
- - + -
hHDAC1 S406E
- - - +
Flag
Histone H3
b
397
406
415
hHDAC1 ...VQMQAIPEDAIPEESGDEDEDDPDKRISICSSDKRIACEEEFSDSDEEGEGGRKNSSNFKKAKR
zfHdac1 ...VQMQAIPEDAVQEDSGDE-EDDPDKRISIRAHDKRIACDEEFSDSEDEGQGGRRNAANYKKPKR

a

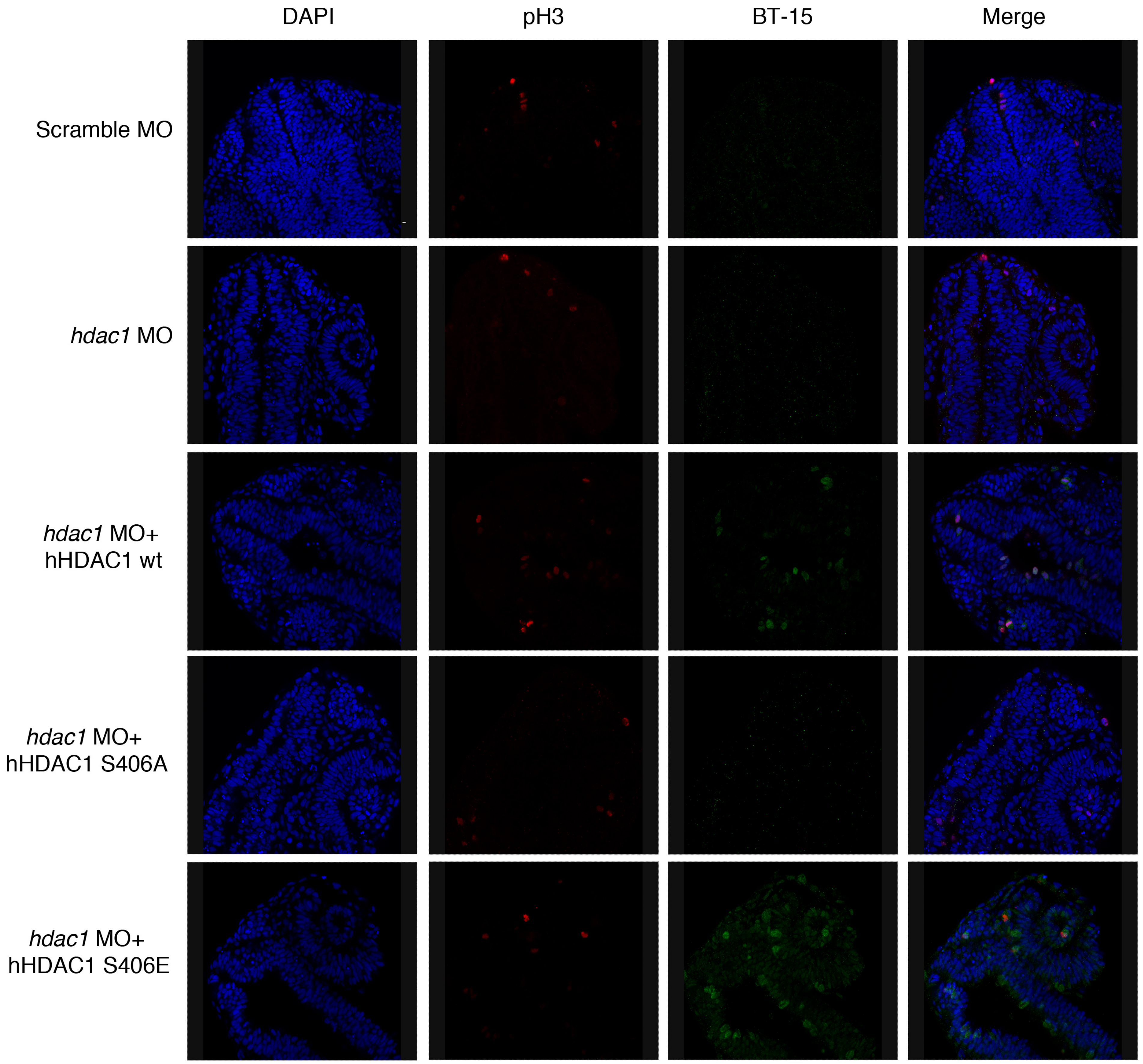
DAPI
pH3
BT-15
Merge
Scramble MO
hdac1 MO
hdac1 MO+
hHDAC1 wt
hdac1 MO+
hHDAC1 S406A
hdac1 MO+
hHDAC1 S406E

b

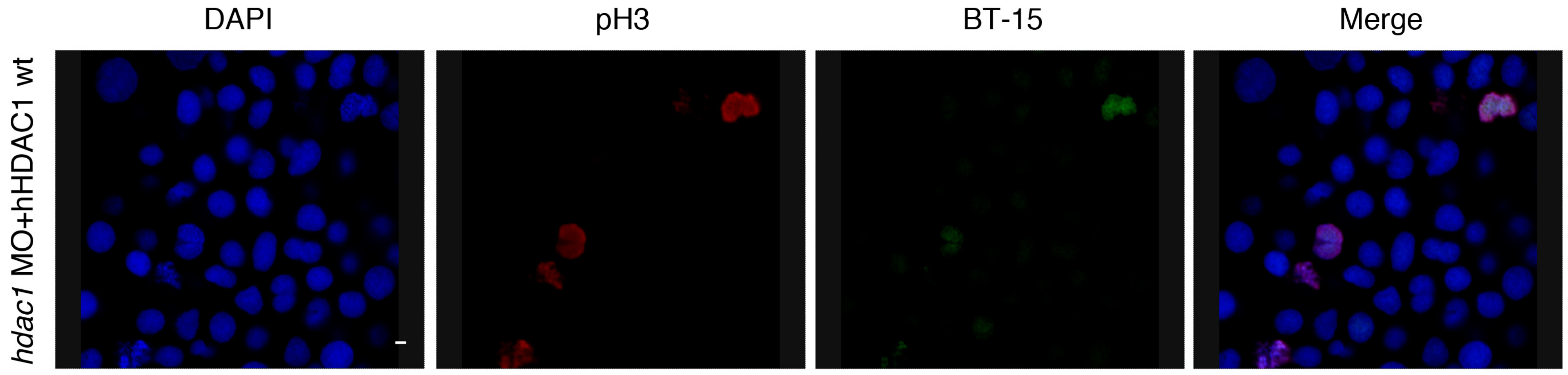
DAPI
pH3
BT-15
Merge
hdac1 MO+hHDAC1 wt

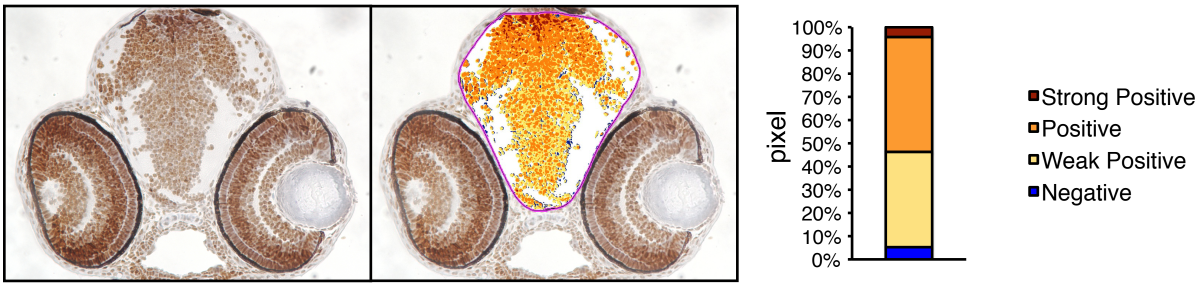

100%
90%
80%
70%
60%
50%
40%
30%
20%
10%
0%
pixel
Strong Positive
Positive
Weak Positive
Negative
